# Adrenalectomies in children and adolescents in Germany – a diagnose related groups based analysis from 2009-2017

**DOI:** 10.3389/fendo.2022.914449

**Published:** 2022-07-27

**Authors:** Konstantin L. Uttinger, Maria Riedmeier, Joachim Reibetanz, Thomas Meyer, Christoph Thomas Germer, Martin Fassnacht, Armin Wiegering, Verena Wiegering

**Affiliations:** ^1^ Department of General, Visceral, Transplant, Vascular and Pediatric Surgery, Würzburg University Hospital, Würzburg, Germany; ^2^ Department of Visceral, Transplant, Thoracic and Vascular Surgery, Leipzig University Hospital, Leipzig, Germany; ^3^ Department of Pediatrics, Ped. Hematology, Oncology and Stem Cell Transplantation, Würzburg University Hospital, Würzburg, Germany; ^4^ Comprehensive Cancer Centre Mainfranken, University of Würzburg Medical Centre, Würzburg, Germany; ^5^ Department of Endocrine Medicine, Würzburg University Hospital, Würzburg, Germany; ^6^ Department of Biochemistry and Molecular Biology , University of Würzburg, Würzburg, Germany

**Keywords:** pediatric, neuroblastoma – diagnosis, therapy, adrenocortical adenocarcinoma, outcome, volume, adrenalectomia

## Abstract

**Background:**

Adrenalectomies are rare procedures especially in childhood. So far, no large cohort study on this topic has been published with data on to age distribution, operative procedures, hospital volume and operative outcome.

**Methods:**

This is a retrospective analysis of anonymized nationwide hospital billing data (DRG data, 2009-2017). All adrenal surgeries (defined by OPS codes) of patients between the age 0 and 21 years in Germany were included.

**Results:**

A total of 523 patient records were identified. The mean age was 8.6 ± 7.7 years and 262 patients were female (50.1%). The majority of patients were between 0 and 5 years old (52% overall), while 11.1% were between 6 and 11 and 38.8% older than 12 years. The most common diagnoses were malignant neoplasms of the adrenal gland (56%, mostly neuroblastoma) with the majority being younger than 5 years. Benign neoplasms in the adrenal gland (D350) account for 29% of all cases with the majority of affected patients being 12 years or older. 15% were not defined regarding tumor behavior. Overall complication rate was 27% with a clear higher complication rate in resection for malignant neoplasia of the adrenal gland. Bleeding occurrence and transfusions are the main complications, followed by the necessary of relaparotomy. There was an uneven patient distribution between hospital tertiles (low volume, medium and high volume tertile). While 164 patients received surgery in 85 different “low volume” hospitals (0.2 cases per hospital per year), 205 patients received surgery in 8 different “high volume” hospitals (2.8 cases per hospital per year; p<0.001). Patients in high volume centers were significant younger, had more extended resections and more often malignant neoplasia. In multivariable analysis younger age, extended resections and open procedures were independent predictors for occurrence of postoperative complications.

**Conclusion:**

Overall complication rate of adrenalectomies in the pediatric population in Germany is low, demonstrating good therapeutic quality. Our analysis revealed a very uneven distribution of patient volume among hospitals.

## Introduction

Adrenal neoplasia are overall rare, especially in childhood. They can arise from the adrenal cortex or the adrenal medulla with different histopathologic origin, distinct differences in dignity (benign/malignant), age distribution, prognosis and hormone production. In addition, in both localizations, several genetic underpinnings have been identified (1–4).

Patients present with a wide range of symptoms, from clinical signs of an unspecific abdominal swelling or recognized symptoms related to hormone overproduction to an incidental finding of an adrenal mass in radiography.

Neoplasms of the adrenal medulla are mostly neuroblastoma followed by pheochromocytoma and other neuroendocrine tumors, that may be found incidentally due to a lack of symptoms but typically present with symptoms such as sweating, flushing, weight loss etc. due to catecholamine excess. In the vast majority of cases, surgical treatment is necessary (1). Since neuroblastoma account for approx. 7% of childhood tumors and have their median age around the age of 2 (5), the majority of infant adrenal tumors can be assigned to neuroblastoma.

Among childhood neoplasms arising from the adrenal cortex, only around 20% are benign adenomas (ACA) with an excellent prognosis, whereas the majority are adrenocortical carcinoma (ACC) with malignant behavior. Pediatric ACC are predominantly hormone secreting and surgical complete resection is the only curative treatment option. Distinction between benign and malign tumors remains challenging (1, 4). There is no known specific age distribution of the tumor entities.

Regardless of the genetic background, age, hormone production and malignant behavior, adrenalectomy is a cornerstone within the multidisciplinary treatment algorithms. However, substantial differences exist between minimally invasive adrenalectomy for small, mostly benign adrenal tumors, and open extended resections for large malignant tumors infiltrating surrounding tissue. Dependent on the surgical approach, there are significant differences regarding postoperative morbidity as well as mortality rates.

For several decades, it has become increasingly obvious that individual surgeon volume and annual hospital case load clearly correlate with patients’ postoperative outcome (6, 7). This has especially been proven for complex cancer surgery in adults (8, 9). Recent data from nationwide analysis also demonstrate a volume – outcome relationship for adrenalectomies of adults (10, 11). To date, it is unclear whether there is also a correlation between hospital volume and patient outcome for adrenal surgery in pediatric patients. Furthermore, no systematic analysis of complication rates and surgical approaches in pediatric patient undergoing adrenal surgery exists.

In this study, we used nationwide billing data to describe a cohort of patients below 21 years undergoing adrenalectomy. Primary outcome was occurrence of postoperative complications by patients’ age, hospital volume as well as main diagnosis leading to adrenalectomy. Secondary outcome was, among others, was age distribution of malignant and non-malignant adrenal neoplasia within this cohort.

## Methods

This is a register based, retrospective nationwide cohort-study on the basis of anonymized diagnose related groups (DRG) billing data provided by the “Statistische Bundesamt” (Federal Statistical Office in Germany) of all adrenal gland resections among children and adolescents performed between 2009 and 2017 in Germany. Hospital reimbursement in Germany is regulated by the DRG-system. Hospitals report the patients diagnoses (primary diagnose as well as potential postoperative complications) their performed medical procedures as well as discharge data to the incurrence. This data are also reported to the Federal Statistical Office.

**Figure 1:**
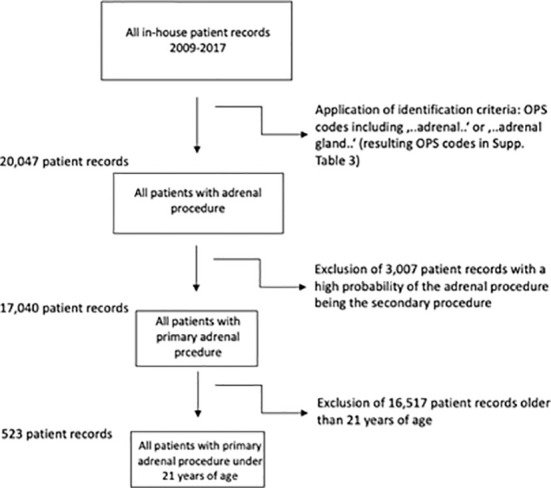
Inclusion criteria and flow-chart. Resulting ICD codes after application of OPS Codes in **Supplementary Table 1**, final ICD codes after exclusion of most likely secondary procedures in **Supplementary Table 2**.

Identification of patients for this study was done using OPS codes including “adrenal…” or “adrenal gland…”. The resulting ICD codes (**Supplementary Table 1**) led to the inclusion of ICD codes (**Supplementary Table 2**) after application of an in-detail bias analysis (ICD codes which were most likely the main diagnosis and led to an accompanying adrenal gland resection were excluded due to possible bias, **Figure 1**). In each individual case, the OPS codes (**Supplementary Table 3**) were hierarchized and the most radical procedure was defined as the main intervention (for procedural hierarchy see **Supplementary Table 4**). Primary nephral diagnoses (like C64, renal cell carcinoma) were not included due to high bias risk.

Relevant complication DRG coding was conducted on the basis of clinical relevance.

Data acquisition was conducted in close contact with the Research Center of the Federal Statistical Office *via* remote analysis (FDZ of the Federal Statistical Offices and the federal states; Data source: Diagnosis-Related Group Statistics (2009-17)) and in accordance with their guidelines for handling highly sensitive patient-record data. Each patient record was assigned an age, gender, an anonymized institute identifier, procedural codes (OPS), main and secondary diagnoses (ICD-10-GM), length of stay and reason for admission and discharge. Duplicates were identified and, in case of occurrence, one data set was chosen randomly to minimize bias. We only analyzed complete data records. ICD-codes for complications were identified among patients’ secondary diagnoses.

All patient records of patients older than 21 years of age were excluded. Before this was done, the same identification criteria were applied to all DRG data and, as a result, all adrenalectomies were primarily identified, regardless of age. This is how this work can present “total number of adrenalectomies performed at the same hospital, including grown-ups” (see in Results section).

Patient records were into three volume tertiles categories of approximately equal size based on their pooled number of adrenal gland resections between 2009 and 2017 (12, 13). For this the entire cohort is divided in three equally sized groups according to the annual hospital volume of pediatric adrenal resections.

Odds ratios (OR) were calculated as risk assessment between the primary dependent variable “hospital complication” and the primary independent variable “dignity” as well as secondary independent variables. A multivariable logistic regression model was used to analyze the relation between hospital tertile and hospital mortality while taking into account possible confounding variables. In order to account for different comorbidity structures, we used the comorbidity score first introduced by Stausberg et al. (14) whose validity has been affirmed in the German variant of the ICD-system. Eligible confounding variables including comorbidity, age and gender were controlled for. Trends were assessed using non parametric trends (15). We excluded the presence of significant multicollinearities among confounding variables. Using the Mantel-Haenszel method, relevant effect modification was checked for.

For the multivariable logistic regression model, the relation between hospital tertile and in-house mortality was determined while accounting for possible confounders and the clustered data structure treating the constant hospital identifier as random effect. Likelihood tests were used to assess the regression model accuracy. Refitting the models for different quadrature points and comparing the values of the estimators helped check the accuracy of the random effects estimators. A resulting maximum difference of ≤ 10^-4^ between the distinct quadrature points was accepted.

Graphs were generated using Prism (Version 9). Stata (Version 16; StataCorp LP, USA) was used for all statistical analysis. P-values of ≤ 0.05 were considered significant.

## Results

A total of 20.047 patients with an adrenal surgical procedure code were (OPS 5-071 for partial and 5-072 for adrenalectomy, among others, details in **Supplementary Figure 3**) reported to the German Federal Statistical Office between 2009 and 2017, of whom 17.040 had a primary adrenal surgery due to a pathology within the adrenal gland (see **Figure 1**). Of these, 523 patients had an age below 21 years. In this cohort, the median age was 8.6 ( ± 7.7) years, 262 patients (50.1%) were female and roughly 56% had a malignant neoplasia of the adrenal gland. In total, 109 hospitals performed at least one adrenal surgery in the observation period, the mean length of stay was 11.9 ( ± 11.7) days, and 174 resection procedures (33.3%) were performed using a minimally invasive (laparoscopic/retroperitoneoscopic) approach.

The most frequent main diagnosis was benign neoplasia of the adrenal gland (n= 152, 29.1%; D350) followed by malignant neoplasia of the adrenal medulla (n=134; 25.6%; C741), malignant neoplasia of the adrenal gland (not otherwise specified) (n=114, 21.8%; C749) and malignant neoplasia of the adrenal cortex (n=43, 8.2%; C740).

When analyzing the patient cohort according to the patient’s age (see also **Table 1**), there were significant differences. With higher age, a clear shift towards female patients (p<0.001), a shorter length of stay (p<0.001) and a higher percentage of minimally invasive procedures (p<0.001) were observed. Also, the rate of malignant adrenal tumors decreased with increasing age (p<0.001).

**Table 1:** Patients ≤21 years of age: patient characteristics by age groups.

z No. of patients between the age of … years	0-1	2-5	6-11	12-17	18-21	P-value ^‡^
n	128 (24.5)	134 (25.6)	58 (11.1)	89 (17.0)	114 (21.8)	
no. of hospitals over the years 33.4 (25-38)	43	38	29	48	70	<0.001
length of stay (days)* 11.9 ±11.7	13.2 (16.8)	13.6 (10.3)	12.0 (8.7)	10.4 (8.1)	9.6 (9.1)	<0.001 n.p.
age in years* 8.6 ±7.7	0.4 (0.5)	3.0 (1.0)	8.5 (1.8)	15.1 (1.6)	19.6 (1.1)	<0.001 n.p.
comorbidity score #_1_ *	101.9 (3.2)	102.7 (4.0)	101.9 (3.8)	99.8 (3.0)	98.7 (3.1)	<0.001 n.p.
minimally invasive procedure n (%)	25 (19.5)	12 (9.0)	13 (22.4)	46 (51.7)	78 (68.4)	<0.001
no. of females 262 (50.1)	54 (42.2)	51 (38.1)	30 (51.7)	49 (55.1)	78 (68.4)	<0.001
total no. of adrenalectomies (including adult patients) performed in same hospital	225.8 (206.0)	285.8 (214.3)	245.1 (222.1)	243.6 (287.5)	221.6 (284.7)	0.005
**Exact diagnosis**						<0.001
C740 (malignant, adrenal cortex) 43 (8.2)	14 (10.9)	13 (9.7)	5 (8.6)	6 (6.7)	5 (4.4)	
C741 (malignant, adrenal medulla) 134 (25.6)	52 (40.6)	52 (38.8)	16 (27.6)	9 (10.1)	5 (4.4)	
C749 (malignant, adrenal gland, n.o.s.) 114 (21.8)	42 (32.8)	56 (41.8)	n.s. (<10)	n.s. (<10)	3 (2.6)	
D350 (benign, adrenal gland) 152 (29.1)	11 (8.6)	7 (5.2)	19 (32.8)	50 (56.2)	65 (57.0)	
D441 (unknown dignity, adrenal gland) 41 (7.8)	n.s. (<10)	6 (4.5)	5 (8.6)	11 (12.4)	11 (9.7)	
other #_2_ (n.s.) 39 (7.5)	n.s. (<3)	0	n.s. (<7)	n.s. (<10)	25 (21.9)	

Left pillar: total numbers and (%). *values are mean (± s.d.); ‡ Chi^2^ test for difference between subgroups. n.p. for non-parametric tests. Details in Supplementary N.s. not stated due to German data law legislation (at least one group is n<3). #_1_ Comorbidity score introduced by Stausberg et al. (14) and validated in the German ICD-10 system. #_2_ ‘other’ combines other resulting main diagnoses (of which each single one was n<20, all ICD codes of the main diagnoses in this study in **Supplementary Table 2**) after application of OPS codes as identification criteria (**Supplementary Table 3**).

As we observed significant differences within the distribution of malignant and non-malignant neoplasia, we divided the cohort accordingly. Patients with a malignant neoplasia were in average 10 years younger (mean 4.2 ± 5.3years; p<0.001)), were more often male (60.9%; p<0.001), had a higher comorbidity score (p<0.001) and a longer length of stay (p<0.001) (details are given in **Table 2**).

**Table 2:** Patients ≤21 years of age: patient characteristics by dignity.

Tumor dignity	malignant	benign	P-value ^‡^
n	294 (56.2)	229 (43.8)	
no. of hospitals over the years (n)	59	93	
length of stay (days) *	13.9 (13.7)	9.3 (7.6)	<0.001 n.p.
age in years *	4.2 (5.3)	14.4 (6.4)	<0.001 n.p.
comorbidity score *	102.4 (3.9)	99.3 (2.8)	<0.001 n.p.
Minimally invasive procedure n (%)	35 (11.9)	139 (60.7)	<0.001
no. of females	115 (39.1)	147 (64.2)	<0.001
average total no. of adrenal resections (including adult patients #_1_) performed in same hospital	229.6 (273.8)	257.7 (217.6)	
**Age groups (years)**			<0.001
0-1 (128, 24.5%)	108 (36.7)	20 (8.7)	
2-5 (134, 25.6%)	121 (41.2)	13 (5.7)	
6-11 (58, 11.1%)	32 (10.9)	26 (11.4)	
12-17 (89, 17.0%)	17 (5.8)	72 (31.4)	
18-21 (114, 21.8%)	16 (5.4)	98 (42.8)	

*values are mean (± s.d.); ‡ Chi^2^ test for difference between subgroups. n.p. for non-parametric tests. #_1_ Adult patients (age >21) were not part of the study cohort but total adrenal gland resection volume per hospital was determined.

As surgery is one cornerstone within the multimodal treatment of patients with adrenal tumors, we analyzed the occurrence of postoperative complications. Overall, more than one quarter had at least one complication, which is mainly directly associated with the surgical procedure like postoperative bleeding. To note, patients with a malignant tumor had a significant risk to suffer from any postoperative complication; especially the need for blood transfusions (approx.40%; p<0.001) was significantly more common than in non-malignant tumors.

To analyze the impact of hospital volume on the postoperative outcome, we divided the entire cohort in three equally sized groups of low, medium and high-volume centers according to the hospital volume of pediatric adrenal resections over the entire study period (details are listed in **Table 3**). This categorization revealed 85 hospitals operated on 31.4% (n=164) of all patients defined as “low volume”. Another 16 hospitals operating an 29,5% (n=154) patients defining as “medium volume” and 8 hospitals operating 39.3% (n=205) of all patients defined as “high volume” This means that, on average, each low volume hospital performed two pediatric adrenal gland resections over the course of 9 years. In addition, the overall caseload of adrenal resections regarding all age groups (including adult patients) in the eight high volume centers was significantly higher than in the low volume centers. Patients in high volume centers were significantly younger (p<0.001), were more likely to have a malignant neoplasia (p<0.001) and underwent more extended (p<0.001) and open resections (p=0.015). For example, while less than 10% of patients underwent an extended resection in a low volume center, approximately one third of patients underwent an extended resection in a high volume center. In line with this differences, more complications occurred in the high volume centers.

**Table 3:** Patients ≤21 years of age: patient characteristics by hospital tertiles.

No. of patients who had surgery in a hospital with a patient volume in the…	low volume tertile	medium volume tertile	high volume tertile	P-value ^‡^
No. of patients (523)	164 (31.4)	154 (29.5)	205 (39.2)	
No. of hospitals in total (33.4 annually on average between 2009 and 2017)	85	16	8	
average total no. of adrenal resections (including adult patients #_1_) performed in same hospital	66.1 (44.9)	182.7 (91.3)	435.9 (281.4)	<0.001 n.p.
Length of stay (11.9 ±11.7)*	10.9 ± 11.6	11.6 ± 9.1	13.0 ± 13.3	0.057 n.p.
Age (8.6 ±7.7)*	11.3 ± 8.0	8.6 ± 7.7	6.5 ± 6.8	<0.001 n.p.
Age groups (years)				<0.001
0-1 (128, 24.5%)	34 (20.7)	38 (24.7)	56 (27.3)	
2-5 (134, 25.6%)	22 (13.4)	42 (27.3)	70 (34.2)	
6-11 (58, 11.1%)	17 (10.4)	12 (7.8)	29 (14.2)	
12-17 (89, 17.0%)	32 (19.5)	31 (20.1)	26 (12.7)	
18-21 (114, 21.8%)	59 (36.0)	31 (20.1)	24 (11.7)	
No. of females (262, 50.1%)	96 (58.5)	74 (48.1)	92 (44.9)	0.028
Comorbidity score (101.0, ±3.8)*	100.1 ± 3.0	100.7 ± 3.2	102.1 ± 4.4	<0.001 n.p.
Exact diagnosis				<0.001
C740 (malignant, adrenal cortex)	13 (7.9)	12 (7.8)	18 (8.8)	
C741 (malignant, adrenal medulla)	29 (17.7)	30 (19.5)	75 (36.6)	
C749 (malignant, adrenal gland, n.o.s.)	25 (15.2)	36 (23.4)	53 (25.9)	
D350 (benign, adrenal gland)	63 (38.4)	52 (33.8)	37 (18.1)	
D441 (unknown dignity, adrenal gland)	13 (7.9	15 (9.7)	13 (6.3)	
other (n.s.)	21 (12.8)	9 (5.8)	9 (4.4)	
Main procedure				0.019
Adrenalectomy (352, 67.3%)	121 (73.8)	100 (64.9)	131 (63.9)	
Partial adrenalectomy (46, 8.8%)	12 (7.3)	8 (5.2)	26 (12.7)	
other (125, 23.9%)	31 (18.9)	46 (29.9)	48 (23.4)	
Main procedure performed was.				0.015
. minimally invasive (174, 33.3%)	69 (42.1)	46 (29.9)	59 (28.8)	
. open (349, 66.7%)	95 (57.9)	108 (70.1)	146 (71.2)	
Overall resection…				<0.001
of adrenal gland only (413, 79.0%)	149 (90.9)	123 (79.9)	141 (68.8)	
with an accompanying resection of other organ(s) (110, 21.0%)	15 (9.1)	31 (20.1)	64 (31.2)	
Overall complication(s) (141, 27.0)	27 (16.5)	32 (20.8)	82 (40.0)	<0.001

Left pillar: total numbers in (). *values are mean (± s.d.); ‡ Chi^2^ test for difference between subgroups. n.p. for non-parametric tests. Details in **Supplementary Table 5**. #_1_ Adult patients (age >21) were not part of the study cohort but total adrenal gland resection volume per hospital was determined.

When analyzing the occurrence of complication, in line with the unequal distribution of age and malignant disease between the hospital volume we observed more frequent overall as well as surgery associated complications within the high-volume centers (**Table 5**).

In a crude analysis, age category, dignity, operative procedure, including minimally invasive and extended resections, and hospital volume were significantly associated with occurrence of postoperative complications (see **Table 4**, **5**). They were therefore considered as potential confounders and included in a regression analysis. In a multivariable regression analysis, accounting for patient clustering within institutions and the effect of confounding variables, a highly significant decrease was found in postoperative complication rate following adrenal resection for patient age (p=0.046 and open procedure (p=0.001), while there was no difference regarding the annual caseload (see **Table 6**).

**Table 4:** Patients ≤21 years of age: complications.

No. of patients	malignant	benign	P-value
n	294 (56.2 of total)	229 (43.8 of total)	
Total of patients with complication(s) 168 (32.1)	138 (46.9)	30 (13.1)	<0.001
…. of which n had surgical complication(s) 154 (29.5)	132 (44.9)	22 (9.6)	<0.001
Bleeding occurrence 100 (19.1)	87 (29.6)	13 (5.7)	<0.001
Transfusion occurrence 129 (24.7)	116 (39.5)	13 (5.7)	<0.001
Relaparotomy occurrence 20 (3.8)	14 (4.8)	6 (2.6)	0.205
…. or other complication(s) 51 (9.8)	39 (13.3)	12 (5.2)	0.002
Endocrine insufficiency (4, 2.8)	n.s.	n.s.	0.207
Artificial respiration > 48h occurrence 19 (3.6)	n.s.	n.s.	0.003
Peritonitis/sepsis occurrence 21 (4.0)	17 (5.8)	4 (1.8)	0.020
Pneumonia occurrence 5 (1.0)	n.s.	n.s.	0.864
Acute Kidney Injury occurrence 11 (2.1)	n.s.	n.s.	0.084
Clostridium difficile occurrence 12 (2.3)	n.s.	n.s.	0.012

(Percentages of column). P values stem from Chi^2^ test for difference between subgroups. N.s. not stated due to German data law legislation (at least one group is n<3).

**Table 5:** Patients ≤21 years of age: complications by patient volume.

No. of patients	low volume tertile	medium volume tertile	high volume tertile	P-value
n (%)	164 (31.4)	154 (29.5)	205 (39.2)	
Total of patients with complication(s) 141 (27.0)	27 (16.5)	32 (20.8)	82 (40.0)	<0.001
…. of which n had surgical complication(s) 127 (24.3)	23 (14.0)	26 (16.9)	78 (38.1)	<0.001
Bleeding occurrence 100 (19.1)	17 (10.4)	19 (12.3)	64 (31.2)	<0.001
Transfusion occurrence 129 (24.7)	28 (17.1)	31 (20.1)	70 (34.2)	<0.001
Relaparotomy occurrence 20 (3.8)	n.s.	n.s.	12 (5.9)	0.070
…. or other complication(s) 48 (9.2)	11 (6.7)	10 (6.5)	27 (13.2)	0.040
Endocrine insufficiency (4, 2.8)	n.s.	n.s.	n.s.	0.905
Artificial respiration > 48h occurrence 19 (3.6)	n.s.	n.s.	15 (7.3)	0.001
Peritonitis/sepsis occurrence 21 (4.0)	4 (2.4)	3 (2.0)	14 (6.8)	0.031
Pneumonia occurrence 5 (1.0)	n.s.	n.s.	n.s.	
Acute Kidney Injury occurrence 11 (2.1)	n.s.	n.s.	7 (3.4)	0.244
Clostridium difficile occurrence 12 (2.3)	4 (2.4)	4 (2.6)	4 (2.0)	0.911

(Percentages of column). P values stem from Chi^2^ test for difference between subgroups. N.s. not stated due to German data law legislation (at least one group is n<3).

**Table 6:** Crude Odds Ratios and multiple regression model.

	Unadjusted OR for in-hospital complication		Multivariable logistic regression model for in-house complication	
	Odds Ratio [95% CI]	P-value	Odds Ratio [95% CI]	P-value
Age (years)				
0-1	1		1	
2-5	1.36 [0.83-2.23]	0.229	1.01 [0.57-1.79]	0.970
6-11	0.55 [0.27-1.10]	0.093	0.37 [0.16-0.86]	0.021
12-17	0.29 [0.15-0.59]	0.001	0.42 [0.18-0.98]	0.046
18-21	0.18 [0.09-0.38]	<0.001	0.40 [0.16-0.98]	0.046
Sex				
female	1		1	
male	1.21 [0.83-1.79]	0.315	0.88 [0.54-1.42]	0.588
Dignity				
benign or unknown	1		1	
malignant	3.99 [2.55-6.23]	<0.001	0.62 (0.31-1.22)	0.167
Procedure				
Adrenalectomy	1		1	
Partial adrenalectomy or other	0.48 [0.21-1.11]	0.085	0.97 [0.75-1.26]	0.825
Surgery was…				
minimally invasive	1		1	
open	6.78 [3.77-12.21]	<0.001	3.38 [1.70-6.71]	0.001
Overall resection…				
with an accompanying resection of other organ(s)	1		1	
of adrenal gland only	0.17 [0.11-0.27]	<0.001	0.30 [0.18-0.52]	<0.001
Comorbidity score (incremental)	1.33 [1.25-1.43]	<0.001	1.21 [1.12-1.30]	<0.001
Hospital Tertile				
low volume	1		1	
medium volume	1.38 [0.79-2.43]	0.259	0.82 [0.43-1.58]	0.559
high volume	3.52 [2.14-5.79]	<0.001	1.78 [0.98-3.22]	0.058

ORs are unadjusted and adjusted and are listed solely for primary dependent variable in-house complication. Overall rho = 3.81xe^-6^ (Confidence Interval 6.05xe^-22^ - 1).

## Discussion

This is the first nationwide analysis for adrenal resections within patients below 21 years. We demonstrate that there are two distinct groups of patients. First, patients with malignant neoplasia who are predominant male and younger. Second, patients with benign adrenal tumors who are more likely female and 6 years or older. The other important observation is, that there are 109 hospitals overall in Germany performing adrenalectomy in patients age blow 21.

The described cohort as well as the age distribution of benign and malignant adrenal tumors are in line with data from several large registries, also demonstrating an unequal distribution of age and dignity of adrenal neoplasia (1, 4, 16).

This uneven age distribution may be due to the fact that pediatric malignant neoplasms of the adrenal gland appear to originate in the fetal zone, and as expected for neoplasms of developing tissue, the median age at diagnosis is very young. This developmental pathway is already well known for neuroblastoma, an embryonal tumor of the autonomic nervous system, but embryonal pathogenesis of adrenocortical carcinoma in early childhood has been discussed by several authors as well. Furthermore, malignant childhood adrenal tumors are often associated with genetic alterations and tumor predisposition syndromes, such as Li-Fraumeni syndrome, leading to early onset of neoplasms (1, 4, 16, 17). Fortunately, the total perioperative mortality rate was below 1.5%. As was expected, patients with malignant neoplasia and younger patients were less likely to undergo minimally invasive resection, and this group also had a significant higher complication rate. These results are in line with billing data of the adult cohort. The aggressive growth of malignant tumors may lead to higher perioperative complications, as, for example, the increased risk of bleeding occurrence, being secondary to the tendency of malignant neoplasms to infiltrate blood vessels and nearby organs (11). Nevertheless, when analyzing billing data for occurrence of complications, some bias has to be taken into account. The available billing data reflect procedures and complications within one hospital stay, but do not identify the exact causality for potential complications. This is the case, for example, when the need of a blood transfusion is a complication of the operative procedure or when patients were initially diagnosed with anemia, for example, due to previous (chemo-)therapy, received a transfusion, and underwent adrenalectomy during the same hospital stay. This should be particularly noted when interpreting the significant higher transfusion rate within patients with malignant neoplasia. Also the high rate of documented “complications” within high volume centers could be explained by the unequal distribution of patients. For example patients within high volume centers are more likely to undergo extended resections due to maligned disease, which per se will bias this point. This is supported by the fact, that when accounting for confounders within the multivariate analyze the Odds ratio for occurrence of complication does not differ according to hospital volume. In light of

Surprisingly, we discovered a highly unequal distribution of hospital case load, which is even more surprising considering adrenal resections are overall rare procedures. In the “low volume” tertile, there was roughly one case below 21 years within five years of the study, whereas high volume centers performed approximately 3 adrenal resections per year. In light of actual recommendations of ENSAT and other international experts, an annual case load of more than 20 adrenalectomies per year is recommended (1, 18–20). It is therefore incomprehensive why some of the patients in the study cohort have not been transferred to a high-volume center. Several studies of adult patients have demonstrated that, especially for adrenal resections, there is a significant volume-outcome correlation. From an economic point of view, high volume goes hand in hand with relevant cost savings (10, 11).

In light of the overall low mortality rate, the “small” sample size and the highly diverse cohort, we did not identify a significant volume – outcome relationship. Nevertheless, it has to be discussed if patients will benefit from a centralization of rare and complex surgical procedures within few, highly competent centers. First, these hospitals should be experienced with adrenal gland tumors as well as be equipped with highly specialized adrenal surgical units, including for pediatric patients.

The main strength of this study is the sample size of more than 500 patients with adrenal resections below 21 years of age, the completeness of data and the adjustment for confounding factors as age, malignant processes and co-morbidity.

Major limitations of this analysis are missing data of individual surgeon volume and individual surgeons’ expertise and surgeons specialty (pediatric/endocrine/general) on the postoperative outcome. Furthermore, no information was available on tumor stage, tumor size, exact histopathological phenotype, hormone production, extension of resection and long-term survival of patients. Another limitation is the lack of readmission data, as the statistics include only individual cases per hospital and readmission is not accounted for. In addition, as stated above, the exact causality for potential complications such as blood transfusions could not be derived from the DRG-data set. Furthermore, the exact diagnosis remained unclear in some patients (especially C749 and D441).

In conclusion, we demonstrated that, overall, surgery for adrenal mass in children and adolescents is a save procedure. There are two groups of patients undergoing an adrenal resection with distinct profiles, younger patients with an underlying malignant disease and older patients who are less likely to suffer from maligned disease. Since tumors of the adrenal gland are overall rare and the dignity of the adrenal mass often remains unclear until definitive histopathological examination, our data suggest a treatment of these patients in few, highly specialized centers.

## Data Availability Statement

The original contributions presented in the study are included in the article/**Supplementary Material**. Further inquiries can be directed to the corresponding author.

## Ethics Statement

Ethical review and approval was not required for the study on human participants in accordance with the local legislation and institutional requirements. Written informed consent from the participants’ legal guardian/next of kin was not required to participate in this study in accordance with the national legislation and the institutional requirements.

## Author Contributions

All authors revised the manuscript and finally approved the manuscript. KU, data acquisition and statistical analysis. VW and AW, study design. MR, AW, and VW, writing the manuscript. MR, JR, TM, CG, MF, and AW, critical discussion and data interpretation. All authors contributed to the article and approved the submitted version.

## Conflict of Interest

The authors declare that the research was conducted in the absence of any commercial or financial relationships that could be construed as a potential conflict of interest.

The handling editor CP declared a past co-authorship with the author MF.

## Publisher’s Note

All claims expressed in this article are solely those of the authors and do not necessarily represent those of their affiliated organizations, or those of the publisher, the editors and the reviewers. Any product that may be evaluated in this article, or claim that may be made by its manufacturer, is not guaranteed or endorsed by the publisher.
