# Supplementary material for: Adrenalectomies in children and adolescents in Germany – a diagnose related groups based analysis from 2009-2017
Source: Front Endocrinol (Lausanne). 2022 Jul 27;13:914449. doi: 10.3389/fendo.2022.914449 (PMC9363694; doi:10.3389/fendo.2022.914449)
Supplement: Supplementary file 1 [file DataSheet_1.docx]

# Appendix

| ICDs: |
| --- |
| A18 Tuberculosis of other organs |
| A39 Infection with meningococci |
| C15 Malignant neoplasm of esophagus |
| C16 Malignant neoplasm of stomach |
| C17 Malignant neoplasm of small intestine |
| C18 Malignant neoplasm of colon |
| C19 Malignant neoplasm of rectosigmoid |
| C20 Malignant neoplasm of rectum |
| C22 Malignant bile duct carcinoma |
| C23 Malignant neoplasm of gallbladder |
| C24 Malignant neoplasm of biliary tract |
| C25 Malignant neoplasm of pancreas |
| C25.4 Malignant neoplasm of pancreas, endocrine part |
| Including Langerhans-islands |
| C25.9 Malignant neoplasm of pancreas, not otherwise specified |
| C48 Malignant neoplasm of retroperitoneal space and peritoneum |
| C49 Malignant neoplasm of other tissue or soft tissue |
| C73 Malignant neoplasm of thyroid gland |
| C74 Malignant neoplasm of adrenal gland |
| C74.0 Malignant neoplasm of adrenal cortex |
| C74.1 Malignant neoplasm of adrenal medulla |
| C74.9 Malignant neoplasm of adrenal gland, not otherwise specified |
| C75 Malignant neoplasm of other endocrine glands and related structures |
| C75.0 Parathyroid gland |
| C75.1 Pituitary gland |
| C75.8 Pluriglandular involvement, not otherwise specified |
| C75.9 Endocrine gland, not otherwise specified |
| C76 Other malignant neoplasm of other or non-specified origin |
| C78 Secondary malignant neoplasm of respiratory and digestive organs |
| C78.6 Secondary malignant neoplasm of the retroperitoneum and peritoneum |
| C79 Secondary malignant neoplasm at other or non-specified location |
| C79.7 Secondary malignant neoplasm of adrenal gland |
| C80 malignant neoplasm with undefined location |
| C97 Neoplasm with multiple primary tumors at different locations |
| D09 Carcinoma in situ; other or in a not specified location |
| D13 Benign neoplasm of other and ill-defined parts of digestive system |
| D13.7 Endocrine pancreas |
| including Islet cell tumor, Islets of Langerhans (Insulinom) |
| D20 Carcinoma in situ in other or non-specified location |
| D21 Benign neoplasm of other or soft tissue |
| D30 benign neoplasm urinary organs |
| D34 Benign neoplasm of thyroid gland |
| D35 Benign neoplasm of other and not otherwise specified endocrine glands |
| D35.0 Adrenal gland |
| D35.1 Parathyroid gland |
| D35.2 Pituitary gland |
| D35.8 Pluriglandular involvement |
| D36 Benign neoplasm in undefined location |
| D37 Neoplasm of uncertain or unknown behaviour of oral cavity and digestive organs |
| D37.7 Pancreas |
| D44 Neoplasm of uncertain or unknown behaviour of endocrine glands |
| excl Pancreas (D37.7) |
| D44.0 Thyroid gland |
| D44.1 Adrenal gland |
| D44.2 Parathyroid gland |
| D44.3 Pituitary gland |
| D44.8 Pluriglandular involvement |
| incl Multiple endocrine adenomatosis |
| D44.9 Endocrine gland, not otherwise specified |
| D48 Neoplasm of uncertain or unknown dignity in other of unspecified location |
| E21 Hyperparathyroidism and other disorders of parathyroid gland |
| E22 Overfunction of pituitary gland |
| E24 Cushing syndrome |
| E24.0 Pituitary-dependent Cushing disease |
| including overproduction of pituitary ACTH, pituitary-dependent hyperadrenocoricism |
| E24.3 Ectopic ACTH syndrome |
| including Cushing syndrome as a result of ACTH producing tumor |
| E24.8 Other Cushing syndrome |
| E24.9 Cushing syndrome, not otherwise specified |
| E25 Adrenogenital disorder |
| E26 Hyperaldosteronism |
| E26.0 Primary hyperaldosteronism |
| inkl Conn syndrome, primary aldosteronism due to adrenal hyperplasia (bilateral |
| E26.9 Hyperaldosteronism, not otherwise specified |
| E27 Other disorders of adrenal gland |
| E27.0 Other adrenocortical overactivity |
| including Overproduction of ACTH, not associated with Cushing disease, premature adrenarche, excluding Cushing syndrome |
| E27.1 Primary adrenocortical insufficiency |
| inkl Addison disease, autoimmune adrenalitis |
| excluding Amyloidosis (E.85), tuberculous Addison disease (A18.7), Waterhouse-Friderichsen syndrome (A39.1) |
| E27.2 Addisonian crisis |
| including adrenal crisis, adrenocortical crisis |
| E27.4 Other and unspecified adrenocortical insufficiency |
| including adrenal haemorrhage or infarction, adrenocortical insufficiency not otherwise specified, hypoaldosteronism |
| excluding adrenoleukodystrophy (Addison Schilder syndrome) (E71.3), Waterhouse-Friderichsen syndrome (A39.1) |
| E27.5 Adrenomedullary hyperfunction |
| including adrenomedullary hyperplasia, catecholamine hypersecretion |
| E27.8 Other specified disorders of adrenal gland |
| including abnormality of cortisol-binding globulin |
| E27.9 Disorder of adrenal gland, not otherwise specified |
| E35 Disorder of adrenal glands with an elsewhere specified disorder |
| P54 Hemorrhage of a newborn |
| Q89 Congenital malformation, incl. of adrenal gland |
| S37.8 Trauma of any pelvic organ; adrenal gland |

**Supp. Table 1: ICD Diagnoses for primary identification**

| ICDs: |
| --- |
| C74 Malignant neoplasm of adrenal gland |
| C74.0 Malignant neoplasm of adrenal cortex |
| C74.1 Malignant neoplasm of adrenal medulla |
| C74.9 Malignant neoplasm of adrenal gland, not otherwise specified |
| C75 Malignant neoplasm of other endocrine glands and related structures |
| C75.1 Pituitary gland |
| C75.8 Pluriglandular involvement, not otherwise specified |
| C75.9 Endocrine gland, not otherwise specified |
| C79.7 Secondary malignant neoplasm of adrenal gland |
| C97 Neoplasm with multiple primary tumors at different locations |
| D35 Benign neoplasm of other and not otherwise specified endocrine glands |
| D35.0 Adrenal gland |
| D35.2 Pituitary gland |
| D35.8 Pluriglandular involvement |
| D44 Neoplasm of uncertain or unknown behaviour of endocrine glands |
| excl Pancreas (D37.7) |
| D44.1 Adrenal gland |
| D44.3 Pituitary gland |
| D44.8 Pluriglandular involvement |
| incl Multiple endocrine adenomatosis |
| D44.9 Endocrine gland, not otherwise specified |
| E22 Overfunction of pituitary gland |
| E24 Cushing syndrome |
| E24.0 Pituitary-dependent Cushing disease |
| including overproduction of pituitary ACTH, pituitary-dependent hyperadrenocorcicism |
| E24.3 Ectopic ACTH syndrome |
| including Cushing syndrome as a result of ACTH producing tumor |
| E24.8 Other Cushing syndrome |
| E24.9 Cushing syndrome, not otherwise specified |
| E25 Adrenogenital disorder |
| E26 Hyperaldosteronism |
| E26.0 Primary hyperaldosteronism |
| inkl Conn syndrome, primary aldosteronism due to adrenal hyperplasia (bilateral |
| E26.9 Hyperaldosteronism, not otherwise specified |
| E27 Other disorders of adrenal gland |
| E27.0 Other adrenocortical overactivity |
| including Overproduction of ACTH, not associated with Cushing disease, premature adrenarche, excluding Cushing syndrome |
| E27.1 Primary adrenocortical insufficiency |
| inkl Addison disease, autoimmune adrenalitis |
| excluding Amyloidosis (E.85), tuberculous Addison disease (A18.7), Waterhouse-Friderichsen syndrome (A39.1) |
| E27.2 Addisonian crisis |
| including adrenal crisis, adrenocortical crisis |
| E27.4 Other and unspecified adrenocortical insufficiency |
| including adrenal haemorrhage or infarction, adrenocortical insufficiency not otherwise specified, hypoaldosteronism |
| excluding adrenoleukodystrophy (Addison Schilder syndrome) (E71.3), Waterhouse-Friderichsen syndrome (A39.1) |
| E27.5 Adrenomedullary hyperfunction |
| including adrenomedullary hyperplasia, catecholamine hypersecretion |
| E27.8 Other specified disorders of adrenal gland |
| including abnormality of cortisol-binding globulin |
| E27.9 Disorder of adrenal gland, not otherwise specified |
| E35 Disorder of adrenal glands with an elsewhere specified disorder |
| Q89 Congenital malformation, incl. of adrenal gland |
| S37.8 Trauma of any pelvic organ; adrenal gland |

**Supp. Table 2: ICD Diagnoses for inclusion**

| **5-071** | **Partial adrenalectomy** |
| --- | --- |
| 5-071.0 | Excision of diseased tissue |
| 5-071.00 | Excision of diseased tissue: open lumbar |
| 5-071.01 | Excision of diseased tissue: open abdominal |
| 5-071.02 | Excision of diseased tissue: open, thoracoabdominal |
| 5-071.03 | Excision of diseased tissue: laparoscopic |
| 5-071.0x | Excision of diseased tissue: not otherwise specified |
| 5-071.4 | Partial adrenalectomy |
| 5-071.40 | Partial adrenalectomy: open lumbar |
| 5-071.41 | Partial adrenalectomy: open abdominal |
| 5-071.42 | Partial adrenalectomy: open, thoracoabdominal |
| 5-071.43 | Partial adrenalectomy: laparoscopic |
| 5-071.4x | Partial adrenalectomy: not otherwise specified |
| 5-071.x | Not otherwise specified |
| 5-071.x0 | Not otherwise specified: open lumbar |
| 5-071.x1 | Not otherwise specified: open abdominal |
| 5-071.x2 | Not otherwise specified: open, thoracoabdominal |
| 5-071.x3 | Not otherwise specified: laparoscopic |
| 5-071.xx | Not otherwise specified: not otherwise specified |
| 5-071.y | not otherwise specified |
| **5-072** | **Adrenalectomy** |
| 5-072.0 | Without ovarectomy |
| 5-072.00 | Without ovarectomy: open lumbar |
| 5-072.01 | Without ovarectomy: open abdominal |
| 5-072.02 | Without ovarectomy: open, thoracoabdominal |
| 5-072.03 | Without ovarectomy: laparoscopic |
| 5-072.0x | Without ovarectomy: not otherwise specified |
| **5-072.1** | **With ovarectomy** |
| 5-072.10 | With ovarectomy |
| 5-072.11 | With ovarectomy: open lumbar |
| 5-072.12 | With ovarectomy: open abdominal |
| 5-072.13 | With ovarectomy: open, thoracoabdominal |
| 5-072.1x | With ovarectomy: laparoscopic |
| 5-072.2 | Adrenalectomy of remaining parts |
| 5-072.20 | Adrenalectomy of remaining parts: open lumbar |
| 5-072.21 | Adrenalectomy of remaining parts: open abdominal |
| 5-072.22 | Adrenalectomy of remaining parts: open, thoracoabdominal |
| 5-072.23 | Adrenalectomy of remaining parts: laparoscopic |
| 5-072.2x | Adrenalectomy of remaining parts: not otherwise specified |
| **5-072.x** | Not otherwise specified |
| 5-072.x0 | Not otherwise specified: open lumbar |
| 5-072.x1 | Not otherwise specified: open abdominal |
| 5-072.x2 | Not otherwise specified: open, thoracoabdominal |
| 5-072.x3 | Not otherwise specified: laparoscopic |
| 5-072.xx | Not otherwise specified: not otherwise specified |
| 5-072.y | not otherwise specified |
| **5-073** | **Other procedures: adrenal gland** |
| **5-073.0** | **Incision, including: with drainage** |
| 5-073.00 | Incision: open lumbar |
| 5-073.01 | Incision: open abdominal |
| 5-073.02 | Incision: open, thoracoabdominal |
| 5-073.03 | Incision: laparoscopic |
| 5-073.0x | Incision: not otherwise specified |
| **5-073.1** | **Plastic reconstruction** |
| 5-073.10 | Plastic reconstruction: open lumbar |
| 5-073.11 | Plastic reconstruction: open abdominal |
| 5-073.12 | Plastic reconstruction: open, thoracoabdominal |
| 5-073.13 | Plastic reconstruction: laparoscopic |
| 5-073.1x | Plastic reconstruction: not otherwise specified |
| **5-073.2** | **Reimplantation of adrenal gland tissue (Autotransplantation)** |
| 5-073.20 | Reimplantation of adrenal gland tissue: open lumbar |
| 5-073.21 | Reimplantation of adrenal gland tissue: open abdominal |
| 5-073.22 | Reimplantation of adrenal gland tissue: open, thoracoabdominal |
| 5-073.23 | Reimplantation of adrenal gland tissue: laparoscopic |
| 5-073.2x | Reimplantation of adrenal gland tissue: not otherwise specified |
| **5-073.4** | **Destruction** |
| 5-073.40 | Using radiofrequency ablation |
| 5-073.41 | Using microwave ablation |
| 5-073.42 | Using irreversible electroporation |
| 5-073.43 | Using electrochemotherapy |
| 5-073.4x | Sonstige |
| **5-073.x** | **Not otherwise specified** |
| 5-073.x0 | Not otherwise specified: open lumbar |
| 5-073.x1 | Not otherwise specified: open abdominal |
| 5-073.x2 | Not otherwise specified: open, thoracoabdominal |
| 5-073.x3 | Not otherwise specified: laparoscopic |
| 5-073.xx | Not otherwise specified: not otherwise specified |
| 5-073.y | not otherwise specified |
| **5-554** | **Nephrectomy** |
| 5-554.4, 40 open lumbar, 41 abdominal, 42 thoracoabdominal, 43 laparoscopic, 4x not otherwise specified | Nephrectomy, radical, including: regional lymphadenectomy, including: adrenalectomy of the same side is included |
| 5-554.5, 50 open lumbar, 51 abdominal, 52 thoracoabdominal, 53 laparoscopic, 5x not otherwise specified | Nephrectomy, radical, with ureterectomy, including: regional lymphadenectomy, including: adrenalectomy of the same side is included |
| 5-554-6, 60 open lumbar, 61 abdominal, 62 thoracoabdominal, 63 laparoscopic, 6x not otherwise specified | Nephrectomy, radical, with endoscopic ureterexhairese, including: regional lymphadenectomy, including: adrenalectomy of the same side is included |

**Supp. Table 3: OPS Codes**

| 1 | Nephrectomy with ureterexhairese, lymphadenectomy with adrenalectomy |
| --- | --- |
| 2 | Nephrectomy with ureterectomy, lymphadenectomy with adrenalectomy |
| 3 | Nephrectomy, lymphadenectomy with adrenalectomy |
| 4 | Adrenalectomy with ovarectomy |
| 5 | Adrenalectomy without ovarectomy |
| 6 | Adrenalectomy, not otherwise specified |
| 7 | Destruction |
| 8 | Partial adrenalectomy |
| 9 | Excision of diseased tissue of adrenal gland |
| 10 | Other excision of adrenal gland, not otherwise specified |
| 11 | Incision, not otherwise specified |
| 12 | Plastic reconstruction |
| 13 | Autotransplantation |

**Supp. Table 4: Procedure hierarchy**

| **No. of patients who had surgery in a hospital with a patient volume in the ...** | **low volume tertile** | **medium volume tertile** | **high volume tertile** | P-value ‡ |
| --- | --- | --- | --- | --- |
| **No. of patients (523)** | 164 (31.4) | 154 (29.5) | 205 (39.2) |  |
| **Main surgery** |  |  |  | 0.012 |
| Nephrectomy, adrenalectomy (16, 3.1%) | 3 (1.8) | n.s. | n.s. |  |
| Adrenalectomy n.o.s. (336, 64.2%) | 118 (72.0) | 97 (63.0) | 121 (59.0) |  |
| Partial adrenalectomy (46, 8.8%) | 12 (7.3) | 8 (5.2) | 26 (12.7) |  |
| Excision at adrenal gland (115, 22.0%) | 26 (15.9) | 43 (27.9) | 46 (22.4) |  |
| Incision at adrenal gland, n.o.s. (10, 1.9) | 5 (3.1) | n.s. | n.s. |  |
| **Procedure type** |  |  |  | <0.001 |
| open, abdominal (305, 58.3%) | 84 (51.2) | 86 (55.8) | 135 (65.9) |  |
| open, lumbar (37, 7.1%) | n.s. | n.s. | 10 (4.9) |  |
| open, thoracoabdominal (7, 1.3%) | n.s. | n.s. | n.s. |  |
| laparoscopic (151, 28.9%) | 67 (40.9) | 44 (28.6) | 40 (19.5) |  |
| other (23, 4.4%) | n.s. | n.s. | n.s. |  |

**Supp. Table 5: Procedural details by hospital volume tertiles.** Left pillar: total numbers in (). ‡ Chi^2^ test for difference between subgroups.
